# Validation of the self-test computerised PROTECT Cognitive Test System for detection of dementia and Mild Cognitive Impairment

**DOI:** 10.64898/2026.07.23.26358814

**Published:** 2026-07-27

**Authors:** Anne Corbett, Kim Idar Giske, Abbie Palmer, Millie Sander, Christine Davis, Kate Stych, Mary O’Leary, Vincent Hayman, Adam Bloomfield, Jon Huntley, Dag Aarsland, Nicolas Castellanos-Perilla, Felipe Botero-Rodriguez, Gerard Griffioen, Mieke Nuytten, Chris Fox, Louise Allan, David Llewellyn, Nicholas Ashton, Hanna Huber, Adam Hampshire, Jeffrey Cummings, Sallie Lamb, Jane Atwell-Thomas, Clive Ballard

## Abstract

Detection and characterisation of dementia and Mild Cognitive Impairment (MCI) is essential for diagnosis and to support recruitment of patients into trials of disease-targeted therapies. Computerised systems offer a means of improving detection in community and primary care settings in a scalable way. This study presents further validation of the self-test PROTECT Cognitive Test System of eight assessments of memory, attention and executive function in 36,941 participants (35,822 healthy, 1046 MCI, 73 dementia). PROTECT shows robust separation of dementia and non-dementia participants ( *p* < 0.001), and good discriminative ability for dementia (Area Under the Curve = 0.966) with 90.90% sensitivity and 87.80% specificity. An optimised detection algorithm robustly identified MCI (P<0.001) and predicted 24-month decline across cognitive domains in both amnestic MCI and non-amnestic MCI phenotypes compared with healthy controls. PROTECT cognitive data also correlated strongly with the plasma biomarkers p-tau217 and Neurofilament Light ( *n* = 46). The system provides a means of improving dementia and MCI detection using self-testing, offering a scalable triage and monitoring tool for clinical pathways and trials.

## Full Text Availability

The license terms selected by the author(s) for this preprint version do not permit archiving in PMC. The full text is available from the preprint server.

